# This I believe: agricultural science and genetics

**DOI:** 10.3389/fgene.2012.00282

**Published:** 2012-12-05

**Authors:** M. S. Swaminathan

**Affiliations:** M S Swaminathan Research FoundationChennai, India

I believe that the current concerns of biosafety and the impact of GMOs on biodiversity will soon give way to an appreciation of the potential benefits that the new genetics can confer on humankind. Agricultural science and genetics together have fed the world and will continue to feed the world.

I was a contemporary of James Watson and Francis Crick at the University of Cambridge, U.K., from 1950 to 1952. I was aware that they were working on the molecular structure of DNA in association with Maurice Wilkins and Rosalind Franklin, as I used to visit the Cavendish Laboratory to attend lectures by Professor Max Perutz. Their publication on the double helix structure of the DNA molecule appeared in *Nature* early in 1953 (Watson and Crick, 1953), while I was at the Genetics Laboratory of the University of Wisconsin, Madison, USA. Since then, I have been following the explosive progress of the science of molecular genetics, which has opened up uncommon opportunities for transferring genes across sexual barriers. I was deeply interested in this development, as my work in the early 1950s related to the transfer of genes in tuber-bearing *Solanum* species for characteristics such as frost tolerance and resistance to the golden nematode, *Heterodera rostochiensis*. The donor for frost tolerance was the species *S. acaule*, from the Lake Titicaca region of Peru-Bolivia, while the donor for golden nematode resistance was *S. polyadenium*. Achieving crosses between these species and *S. tuberosum* was extremely difficult, as the foreign pollens were getting inhibited in the stigma and style. To overcome this, I had to remove the stigma and replace it with an artificial medium that promoted pollen germination (Swaminathan, 1955). This technique later came to be known as the “Swaminathan artificial stigma method.”

All this effort would not have been necessary had the recombinant DNA technology existed then. Thus, the Watson–Crick–Wilkins–Franklin discovery paved the way for fulfilling the dream of plant and animal breeders and microbiologists with respect to producing novel genetic combinations of applied interest. The 1958 awarding of the Nobel Prize to my colleague at the University of Wisconsin Dr. Joshua Lederberg, for his work on microbial transformation further stimulated my interest in this fast-growing area of science^1^. In 1982, the US National Academy of Sciences invited me to deliver a lecture on “Biotechnology Research and Third World Agriculture,” where I emphasized the need for Third World countries, to master all recent developments in science (Swaminathan, 1982).

In 1980, when I joined India's Planning Commission at the invitation of then Prime Minister Indira Gandhi, I set up a National Biotechnology Board to achieve synergy and coordination among the work in progress in molecular genetics and genetic engineering under the umbrella of different scientific organizations, including the Indian Council of Agricultural Research (ICAR), the Council of Scientific and Industrial Research (CSIR), the Indian Council of Medical Research (ICMR), the Department of Atomic Energy (DAE), and the University Grant Commission (UGC). I served as the first chair of the board, which was converted into a Department of Biotechnology during the tenure of Prime Minister Rajiv Gandhi, with Dr. S. Ramachandran serving as its first Secretary.

During the last 30 years, the government of India has invested a considerable amount of money in creating the infrastructure essential for advanced research in biotechnology in general and in genomics and genetic engineering in particular. The government of India is also hosting the International Centre for Genetic Engineering and Biotechnology (ICGEB) in New Delhi. Both in India and abroad, much investment has been made in human resource development in the areas of environmental, medical, industrial, food, and agricultural biotechnology. The first patent for a living genetically modified organism was granted to Dr. Anand Chakraborty, in the USA, for his work on the development of a *Pseudomonas* strain that can clean up oil spills. Genetic medicine, including vaccine development, is also making rapid progress. Bioremediation is gaining in importance with the growing pollution of water. However, in the field of agricultural and food biotechnology, there are concerns about biosafety, environmental safety, biodiversity loss, and food safety. The Global Biodiversity Convention, adopted at Rio de Janeiro in June 1992, has the following clauses with respect to biotechnology:

“Each Contracting Party shall take legislative, administrative or policy measures, as appropriate, to provide for the effective participation in biotechnological research activities by those Contracting Parties, especially developing countries, which provide the genetic resources for such research, and where feasible in such Contracting Parties.”

“The Parties shall consider the need for and modalities of a protocol setting out appropriate procedures, including, in particular, advance informed agreement, in the field of the safe transfer, handling and use of any living modified organism resulting from biotechnology that may have adverse effect on the conservation and sustainable use of biological diversity.”

This resulted in the adoption of the Cartagena Protocol for biosafety. The Cartagena Protocol on Biosafety to the Convention on Biological Diversity is the only international environmental agreement that is concerned exclusively with the transboundary movement (i.e., trade) of products of modern biotechnology that are living modified organisms. It applies to the transboundary movement, transit, handling, and use of all living modified organisms that may have adverse effects on the conservation and sustainable use of biological diversity, taking into account risks to human health. GM foods are considered only if they are LMOs that may be subject to transboundary movement for direct use as food, feed, or for processing. The protocol does not apply to processed food products, nor does it address the food safety of LMOs that are for food, feed, or processing.

I am confident that the science of molecular genetics developed during the last 60 years will not only help humankind to face the challenges to sustainable food security arising from climate change, but will also help to overcome diseases such as Parkinson's, Alzheimer's, and cancer. Thus, the use of the tools of molecular genetics based on a careful and transparent analysis of the risks and benefits by a competent regulatory agency will help us to enter into an era of biohappiness based on the intelligent application of genetics to human well-being.

## Footnotes

^1^Dr. Joshua Lederberg, Nobel Laureate in Medicine and Physiology: “I can think of no one who has combined the insights of the cutting edges of biological science, with attention to the most urgent of human needs, with the competence, devotion, and energy that Dr. M. S. Swaminathan has given. The world is and will be a better place on account of his contributions to its welfare” (Dil, 2004).
